# An Unusual Case of Choledochal Cyst

**DOI:** 10.1055/s-0038-1673379

**Published:** 2018-10-18

**Authors:** Manish Pathak, Rahul Saxena, T.K. Jayakumar, Arvind Sinha, Taruna Yadav

**Affiliations:** 1 Department of Pediatric Surgery, All India Institute of Medical Sciences, Jodhpur, Jodhpur, Rajasthan, India; 2 Department of Diagnostic and Interventional Radiology, All India Institute of Medical Sciences, Jodhpur, Jodhpur, Rajasthan, India

**Keywords:** choledochal cyst, cystic biliary atresia, atretic variant

## Abstract

Choledochal cyst (CC) is an important surgical cause of jaundice in infants. Infantile variant of CC can mimic biliary atresia in clinical presentation. CC associated with biliary atresia is well described in literature. We encountered an atretic variant of CC that has not been described in literature ever. The characteristics of this unusual case, management, and proposed hypothesis to its pathogenesis are discussed here.

## Introduction

Biliary atresia and choledochal cyst (CC) are two of the most common causes of obstructive jaundice in infants. Infantile variant of CC can mimic cystic biliary atresia in clinical presentation leading to diagnostic dilemma. 1 2 3 Biliary atresia associated with CC is also reported in literature. Here, we present an unusual case of CC that does not fit into the current classification of CCs, and can be called an atretic variant of CC.

## Case Report

A 73-day-old boy was brought to our hospital with the complaint of progressively increasing jaundice. This boy was conceived by in vitro fertilization, the second among twins, through cesarean section. Antenatal check-ups were unremarkable. Until 15 days of life, this boy was healthy, taking breastfeeds well and passing yellow/greenish stool. Later he developed symptoms: passing clay colored stool, yellowish discoloration of sclera and body. Initial evaluation done in other hospitals revealed cholestatic jaundice. Finally, when the patient was brought to our hospital, he was deeply icteric. The patient was underweight compared with his elder twin. Liver, with smooth margin and soft consistency, was palpable 3 cm below costal margin. Patient had elevated liver enzymes (AST–64 U/L, ALT–129 U/L, ALP–1,000 U/L) and hyperbilirubinemia (Total bilirubin–8.74 mg /dL, direct–4.9 mg/dL). Gamma glutamyl transpeptidase (GGT) was elevated (1,006 U/L). Hemogram, PT/INR, C-reactive protein, and thyroid profile were normal.

**Fig. 1:**
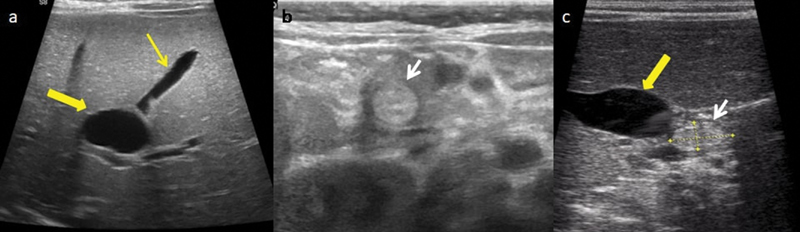
( **a** , **b** , and **c** ) Ultrasound images showing a well-defined gall bladder (thin yellow arrow), dilated common bile duct (CBD; thick yellow arrow), an echogenic calculus in lower CBD (white arrow).

Ultrasonography of abdomen revealed dilated intrahepatic biliary radicles with dilated common bile duct (CBD) till mid part of CBD. A hyperechoic soft calculus without distal acoustic shadowing was seen in the lumen of distal intrapancreatic CBD. Gall bladder (GB) was normal in size with well-defined walls ( Fig. 1 ).

**Fig. 2:**
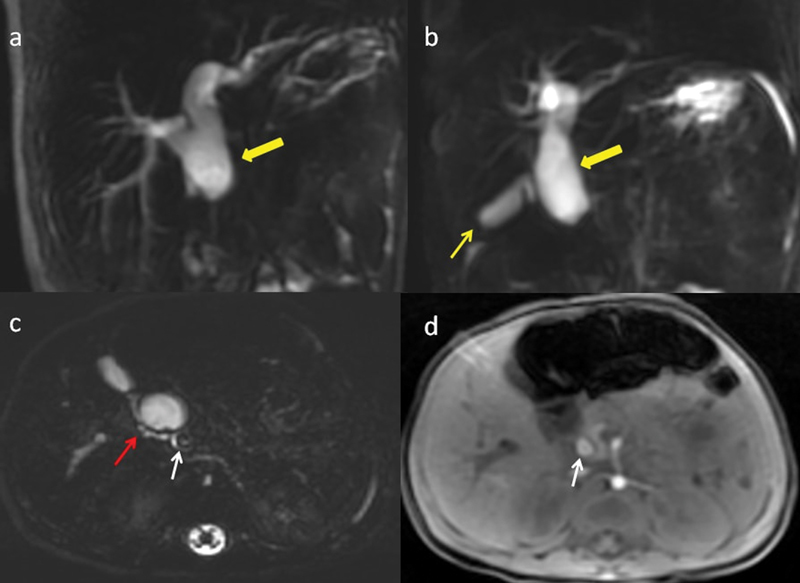
MRI images of a 3-month-old infant with cholestatic jaundice. ( **a** , **b** ) 2D MRCP showing cystic dilatation of common hepatic duct (thick yellow arrow), and gall bladder (thin yellow arrow). ( **c** , **d** ) Axial 3D MRCP and axial T1-weighted MR images depicting course and drainage of cystic duct (red arrow) unusually distally and continuing as CBD which shows a T2 hypointense ( **c** ), T1 hyperintense ( **d** ) calculus (white arrow) within it respectively. 2D, two dimensional; 3D, three dimensional; CBD, common bile duct; MRCP, Magnetic resonance cholangiopancreatography; MRI, magnetic resonance imaging.

**Fig. 3:**
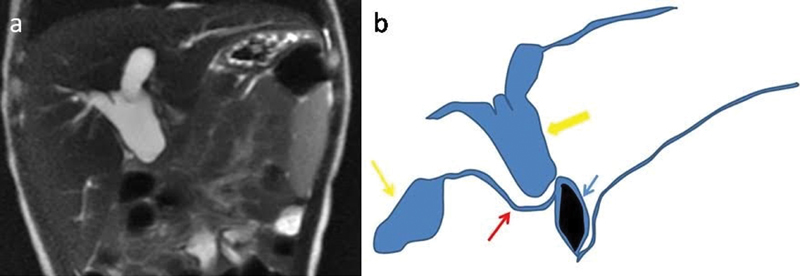
( **a** ) 2D MRCP showing blind ending dilated common hepatic duct. Line diagram ( **b** ) depicting the final preoperative diagnosis of infantile choledochal cyst associated with atresia of common hepatic duct distally resulting in dilated common hepatic duct proximally (thick yellow arrow), normal size of GB (thin yellow arrow), cystic duct insertion in lower part (red arrow) and a calculus in lower CBD (blue arrow), as correlated with Coronal T2-weighted MRI image. CBD, common bile duct; GB, gall bladder; MRI, magnetic resonance imaging.

Further, Magnetic resonance cholangiopancreatographic (MRCP) imaging revealed tubular cystic dilatation of common hepatic duct and confirmed intrahepatic biliary radicles dilatation. Cystic duct was unusually draining quite distally. Abrupt narrowing was seen at the junction of common hepatic duct (CHD) and CBD which was hypothesized to be due to a possible stricture at this level. A calculus (8 mm) was evident in the lower CBD extending into intrapancreatic part of CBD. Cystic duct was draining just above the calculus ( Figs. 2 and 3 ). Main pancreatic duct was not dilated.

HIDA (hepatobiliary iminodiacetic acid scan) scan revealed no radioisotope excretion into gut even after 24 hours. Presumptive diagnosis of choledochal cyst was made before taking up the patient for laparotomy.

**Fig. 4:**
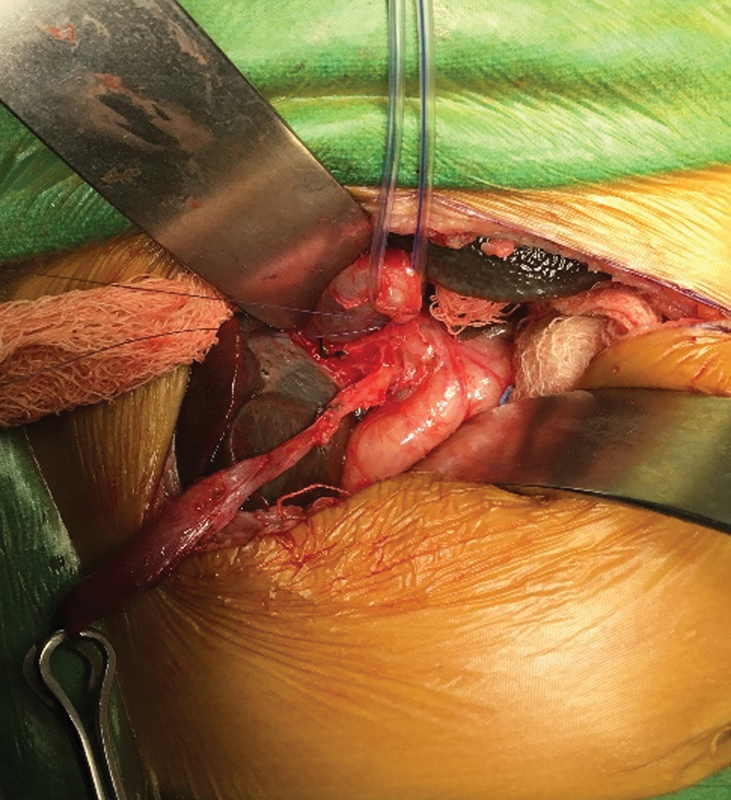
Intraoperative image showing normal sized gall bladder, dilated common hepatic duct (cyst) and cystic duct noncommunicating with common hepatic duct.

**Fig. 5:**
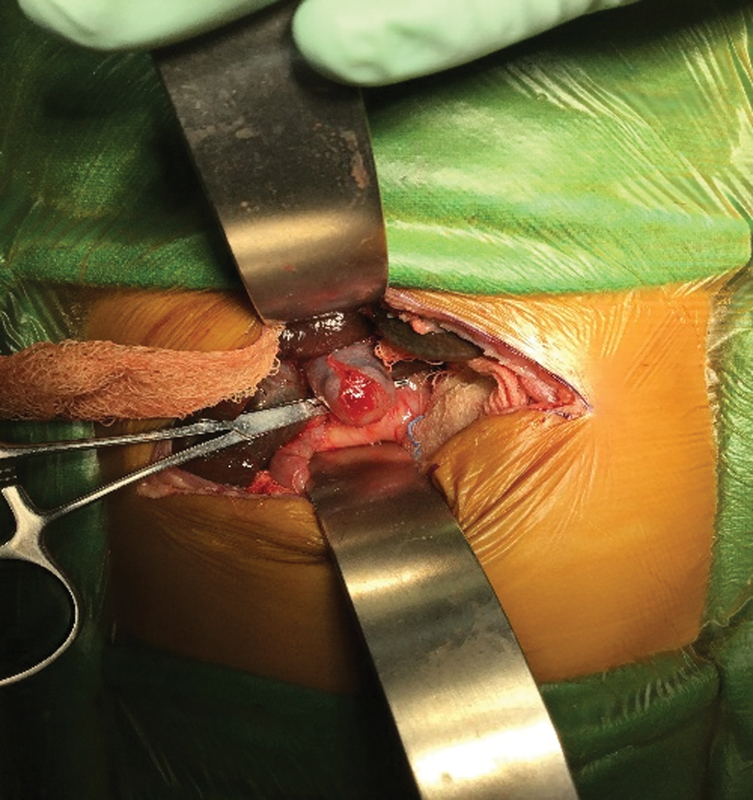
Intraoperative image showing blind ending dilated common hepatic duct (cyst) noncommunicating with cystic duct.

Intraoperatively, hepatomegaly normal size GB, and a 3 × 2.5 cm cyst in subhepatic region were found ( Fig. 4 ). Bile was seen on needle aspiration from cyst. Mobilization of GB was done and cystic duct was found to be opening into duodenum, without any communication with the cyst or CHD. The cyst was mobilized and looped with a feeding tube. On further dissection, cyst was found to have a blind ending distally ( Fig. 5 ). Right and left hepatic ducts were patent and opening into the cyst. Both hepatic ducts were irrigated with saline. Excision of cyst and hepaticodocho-jejunostomy was done. Distally, ligation and division of cystic duct at the entry into duodenum, and cholecystectomy completed the procedure. Postoperatively oral feeds were started after 48 hours. Patient passed normal colored stool and showed normal weight gain. Jaundice had subsided and he was discharged after 5 days.

Histopathological examination of cyst (cuboidal epithelium, stromal mononuclear infiltrates) and liver (normal lobular architecture and no evidence of fibrosis, giant cells) were consistent with choledochal cyst.

Follow-up at 6 weeks was done. The patient was healthy, anicteric, taking feeds well, passing normal colored stool, and gained weight.

## Discussion

Todani modification of Alonso-Lej classification classifies CC into five types ( Table 1 ). 3 CC in infants needs to be differentiated from cystic biliary atresia due to their similar clinical presentation. 2 Nondilatation of intrahepatic biliary radicles and absence of biliary epithelial lining of cyst; differentiates cystic biliary atresia from CC in infants. 1 2 Contrary to CC, cystic biliary atresia does not have any continuity with biliary tree and aspirate of cyst is nonbilious. HIDA scan in cystic biliary atresia does not reveal any excretion of radioisotope into the gut. Our case had radiological, intraoperative and histopathological findings of CC but the unusual intraoperative finding was the completely atretic distal end of CC and noncommunication of cystic duct with the cyst. This finding does not fit into any of the present classification of CC. 3 4 Though distal stenosis of CC and association of CC with biliary atresia are well described in literature, an atretic variant of CC is not yet described, to the best of our knowledge.

**Table 1:** Todani modification of Alonso-Lej classification for choledochal cyst

Type I	Fusiform or cystic dilatation of CBD
Type II	Bile duct diverticulum
Type III	Choledochocele
Type IV	Multiple extrahepatic and intrahepatic dilation of duct IV a both intra and extrhepatic ducts are affected IV b involves only extraheptic bile duct
Type V	Fusiform or saccular dilation of intrahepatic bile ducts (Caroli disease)

This new entity also raises curiosity to its embryological basis. Various hypotheses have been proposed to explain the development of CC. Most often cited theory is anomalous pancreaticobiliary junction with reflux of pancreatic juice into the bile duct causing weakness and dilatation of duct wall. 5 Other theories include distal stenosis, sphincter of oddi dysfunction, faulty remodeling of the embryonic ductal plate, motility disorders and viral damage to the ganglion cells of bile duct. 4 5 6 Our case was not associated with other congenital anomalies so the inciting insult was probably late perinatal event instead of early embryological one which is usually associated with congenital anomalies of other organs developing at the same time. 7 The finding of distal atresia of the bile duct with discontinuity of cystic duct from bile duct is similar to the type-3 jejunoileal atresia (JIA). It is widely accepted that jejunoileal atresia results from vascular insult late in foetal life. Similar to JIA, late perinatal ischemic insult can best explain the pathogenesis of this atretic variant of CC. 8 9

## Conclusion

Atretic variant of CC is a newly described entity that can mimic cystic biliary atresia and other variants of CC in clinical presentation. It requires early surgical intervention to prevent irreversible changes in the liver. In addition, this variant is expected to behave like other variants of CC with excellent prognosis if timely intervention is done.

## Notes

New Insights and the Importance for the Pediatric Surgeon An atretic variant of choledochal cyst is described as a variant that needs early surgical intervention to prevent irreversible damage to liver.
